# Supplementary material for: Gut Microbiome in a Russian Cohort of Pre- and Post-Cholecystectomy Female Patients
Source: J Pers Med. 2021 Apr 12;11(4):294. doi: 10.3390/jpm11040294 (PMC8070538; doi:10.3390/jpm11040294)
Supplement: Supplementary file 1 [file jpm-11-00294-s001.zip › jpm-11-00294-s001.pdf]

Journal of Personalized Medicine

Table 4. Statistical analyses' results: contribution of bacterial families into the principal components extracted from the matrix with relative abundance in feces of females with gallstone disease before the CCE (percentage of total variance in brackets) and *p*-values for multiple regression with age, BMI and blood biochemistry (coefficients of determination in brackets).

**Families: PCA[1]**				
**Main contributors**	**PC 1 (34%)**	**PC 2 (23%)**	**PC 5 (6%)**	**PC 10 (1%)**
Ruminococcaceae	**0.18**[2] [-0.62][3]	0.05	0.00	0.00
Lachnospiraceae	0.09	0.07	0.02	0.02
Enterococcaceae	**0.71** [0.96]	0.02	0.00	0.00
Bifidobacteriaceae	0.01	0.01	**0.81** [0.92]	0.01
Veillonellaceae	0.00	0.00	0.01	**0.36** [-0.51]
Streptococcaceae	0.00	0.00	**0.05** [-0.29]	0.04
Erysipelotrichaceae	0.00	0.00	0.02	**0.48** [0.65]
**Families: Multiple regression**				
R /R^2	0.67 /0.45	0.58 /0.34	0.50 /0.25	0.79 /0.63
Age	***0.03***[4]	0.93	*0.07*	***0.02***
BMI	*0.06*	0.68	0.34	0.89
Glucose	0.32	0.17	0.99	***0.00***
ALT	0.78	0.22	0.11	***0.00***
AST	0.73	0.56	*0.10*	***0.00***
Bilirubin	0.24	0.88	0.25	0.20

[1] PCA stands for principle components analysis (based on covariance). Only those principal components that a) account for the bigger fraction of the total data variance and/or b) displayed statistically significant correlation with patients' characteristic are shown.

[2] The values in bold show the two topmost contributions.

[3] Factor loadings for variables (taxon relative abundance) are given in square brackets.

[4] The values in bold italics and underlined italics are at P≤0.05 and P≤0.10, respectively.
